# Comparative molecular field analysis and molecular dynamics studies of the dopamine D_2_ receptor antagonists without a protonatable nitrogen atom

**DOI:** 10.1007/s00044-018-2137-5

**Published:** 2018-02-13

**Authors:** Agnieszka A. Kaczor, Justyna Żuk, Dariusz Matosiuk

**Affiliations:** 1 Department of Synthesis and Chemical Technology of Pharmaceutical Substances with Computer Modelling Lab, Faculty of Pharmacy with Division of Medical Analytics, Medical University of Lublin, 4A Chodźki St., 20093 Lublin, Poland; 2 School of Pharmacy, University of Eastern Finland, Yliopistonranta 1, P.O. Box 1627, 70211 Kuopio, Finland

**Keywords:** Dopamine D_2_ receptor, Dopamine D_2_ receptor antagonists, CoMFA, QSAR, Molecular modeling, Non-basic compounds.

## Abstract

The dopaminergic hypothesis of schizophrenia is the main concept explaining the direct reasons of schizophrenia and the effectiveness of current antipsychotics. All antipsychotics present on the market are potent dopamine D_2_ receptor antagonists or partial agonists. In this work we investigate a series of dopamine D_2_ receptor antagonists which do not fulfill the criteria of the classical pharmacophore model as they do not possess a protonatable nitrogen atom necessary to interact with the conserved Asp(3.32). Such compounds are interesting, inter alia, due to possible better pharmacokinetic profile when compared to basic, ionizable molecules. By means of homology modeling, molecular docking and molecular dynamics we determined that the compounds investigated interact with Asp(3.32) via their amide nitrogen atom. It was found that the studied compounds stabilize the receptor inactive conformation through the effect on the ionic lock, which is typical for GPCR antagonists. We constructed a CoMFA model for the studied compounds with the following statistics: *R*^2^ = 0.95, *Q*^2^ = 0.63. The quality of the CoMFA model was confirmed by high value of *R*^2^ of the test set, equal 0.96. The CoMFA model indicated two regions where bulky substituents are favored and two regions where bulky substituents are not beneficial. Two red contour regions near carbonyl groups were identified meaning that negative charge would be favored here. Furthermore, the S-oxide group is connected with blue contour region meaning that positive charge is favored in this position. These findings may be applied for further optimization of the studied compound series.

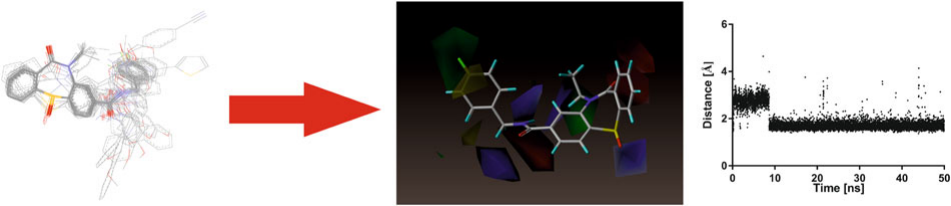

## Introduction

G protein-coupled receptors (GPCRs) account for approximately 30% of all current drug targets (Overington et al. 2006; Komatsu 2015). The dopamine receptors belong to monoamine GPCRs and they play a significant role in the pathophysiology and treatment of psychosis and movement disorders. In particular, multiple psychomotor signs and symptoms are affected by the activity of dopaminergic neurons and by drugs that selectively interact with neuronal dopamine receptors (Missale et al. 2010). These symptoms include rigidity in Parkinson’s disease, dyskinesia in Huntington’s disease, spontaneous oral dyskinesia in the elderly and hallucinations in Alzheimer’s disease and schizophrenia (Missale et al. 2010).

The causes of schizophrenia and—as a consequence—the mechanism of action of antipsychotic drugs are currently not adequately understood. The dopaminergic hypothesis of schizophrenia is the main concept explaining the direct reasons for schizophrenia and the effectiveness of current antipsychotics. According to this hypothesis the pathomechanism of schizophrenia is attributed to the dysfunction of dopaminergic receptors in the mesolimbic system (positive symptoms) and mesocortical pathway (negative symptoms).

Dopamine D_2_ receptor antagonists are used as antipsychotics. Starting from the discovery of the antipsychotic activity of chlorpromazine in 1952, all currently known antipsychotics exhibit affinity for dopamine D_2_-like receptors as an apparently essential aspect of their mechanism of action (Mailman and Murthy 2010), which is in accordance with the dopaminergic hypothesis of schizophrenia. This includes the third generation antipsychotics, aripiprazole, brexpiprazole and cariprazine. The activity of these drugs have been assigned to either D_2_ receptor partial agonism (dopamine receptor stabilizers) or D_2_ receptor functional selectivity (Mailman and Murthy 2010). However, a significant percentage of schizophrenia patients do not respond well to the available treatments. There are two other main limitations of current antipsychotics. Firstly, they are considerably efficient against positive symptoms of schizophrenia (e.g., hallucinations, delusions, and thought disorders) whereas they do not address well negative (e.g., anhedonia and social withdrawal) and cognitive symptoms (e.g., memory and attention deficits) of the disease. Secondly, in particular first generation antipsychotics, exert severe side effects, including neurological disorders termed as extrapyramidal symptoms, sexual dysfunctions, and deterioration of cognitive abilities. Second generation antipsychotics often have metabolic effects leading to weight gain. Thus, novel types of potential drugs against schizophrenia are currently under investigation.

All aminergic GPCRs and some other rhodopsin-like GPCRs, e.g., opioid receptors, share the negatively charged and conserved aspartate residue (D3.32) in the transmembrane helix 3 (TM3) which was generally proposed as a key anchor for the basic moieties of aminergic ligands (Shi and Javitch 2002; Surgand et al. 2006; Kooistra et al. 2013). Accordingly, most dopamine D_2_ receptor orthosteric ligands, including both agonists and antagonists fulfill the criteria of the classical pharmacophore model. The key element of this model is a protonatable nitrogen atom which is able to interact with the conserved Asp(3.32). A positively charged group is also a key element of the pharmacophore model recently constructed for the dopamine D_2_ receptor antagonists (Ekhteiari Salmas et al. 2016). It was found that common pharmacophore motives for the dopamine D_2_ receptor antagonists include AADPR, AADRR, AAHPR, AAPRR, ADHRR, ADPRR, AHHPR, AHHRR, AHPRR, and HHPRR (“A”—hydrogen bond acceptor, “D”—hydrogen bond donor, “H”—hydrophobic group, “P”—positively charged group, and “R”—aromatic ring), however the highest correlation coefficients for training set and test set compounds were found as 0.95 and 0.75, respectively at the AADPR.671 model. This work also indicated other top-ranked 3D QSAR hypotheses (AADRR.1398, AAPRR.3900, and ADHRR.2864) and two of them did not contain a positively charged group. This is in accordance with a few reports which have proven that the presence of a basic nitrogen atom enabling formation of the interaction of its protonated form and D3.32 is not indispensable for the dopamine D_2_ receptor anchoring (Xiao et al. 2014; Kaczor et al. 2016b). Non-basic ligands are also known for some other aminergic GPCRs, e.g., the serotonin 5-HT_6_ receptor (Ivachtchenko et al. 2010) and for opioid receptors, including κ opioid receptor ligand, salvinorin A and μ opioid receptor ligands, carbonyl derivatives of 1-aryl-2-iminoimidazolidine (Matosiuk et al. 2001, 2002a, b; Sztanke et al. 2005).

The development of non-basic, non-ionizable ligands for the treatment of central nervous system (CNS) diseases may be an important improvement taking into account the pharmacokinetics of a potential drug. The most important barrier for drug permeation is due to many lipid barriers which separate body compartments. The ionization state of the drug is an important factor as all the charged drugs diffuse through lipid environments with difficulty. The charged forms of drugs are aqueous-soluble and relatively lipid-insoluble so they does not pass biological membranes easily. pH and the drug pK_a_ are crucial for determination of the ionization state and will significantly influence drug transport. As most drugs are weak acids and/or bases, knowledge of the dissociation constant can help in understanding the ionic form a compound will take across a range of pH values (Manallack 2008). This is especially crucial in physiological systems where ionization state will influence the rate at which the molecule is able to diffuse across membranes and obstacles such as the blood-brain barrier (BBB) (Manallack 2008). The pK_a_ of a drug affects lipophilicity, solubility, protein binding and permeability which—as a consequence—directly influences pharmacokinetic (PK) features, like absorption, distribution, metabolism, and excretion (ADME) (Manallack 2008).

As it was already mentioned, Xiao et al. (2014) obtained a series of the dopamine D_2_ receptor antagonists without a protonatable nitrogen atom. Such ligands are worth detailed investigation as they may exhibit a unique pharmacological profile and may turn out better antipsychotics as well as may be developed as drugs with better pharmacokinetic properties. In this context, we studied the interactions of these dopamine D_2_ receptor antagonists without a protonatable nitrogen atom with the dopamine D_2_ receptor by means of homology modeling, molecular docking and molecular dynamics and we constructed a CoMFA model for them which can enable further modifications within this series of compounds.

## Material and methods

### Homology modeling

The homology model of the human dopamine D_2_ receptor (P14416) in inactive conformation and in complex with an antagonist eticlopride was constructed as previously described (Kaczor et al. 2016a, b, c). The X-ray structure of the dopamine D_3_ receptor in complex with an antagonist eticlopride (PDB ID: 3PBL) (Chien et al. 2010) was used as a template as it is currently the template with the highest sequence identity and similarity to the dopamine D_2_ receptor target. Multiple sequence alignment of 50 rhodopsin-like GPCRs was carried out with the GPCR module of MOE Molecular Environment and refined manually, in particular to satisfy disulfide bridges which were not automatically identified by the software. The dopamine D_2_ receptor model was built without N-terminus (the first 36 residues were removed, the model starts with Tyr37), and without the intracellular loop 3 (ICL3, residues Arg217-Lys362 were removed). Homology modeling was performed using Modeler v.9.10 (Webb and Sali 2014). A hundred homology models of the dopamine D_2_ receptor in complex with eticlopride were generated, and subsequently assessed by Modeler objective function and discrete optimized protein energy profiles (Shen and Sali 2006). The best model was subjected to quality assessments using the Schrödinger suite of software tool for Ramachandran plots.

### Compound preparation

The investigated compounds (the reference ligand chlorprothixene and compounds **1**–**44** were modeled using the LigPrep protocol from the Schrödinger Suite. In order to sample different protonation states of ligands in physiological pH, Epik module was used. The compounds were further optimized with the Wavefunction Spartan10 software. The procedure involved geometry optimization performed with B3LYP DFT using the 6-31G(d,p) basis set.

### Molecular docking

Molecular docking was performed using Glide from the Schrödinger suite of software. The grid file for chlorprothixene docking was generated at default settings, indicating eticlopride as a reference ligand. The complex of the dopamine D_2_ receptor with chlorprothixene was constructed using the SP (standard precision) protocol of Glide. The complex was further refined with induced-fit docking of Schrödinger suite of software and used for grid generation with default settings indicating chlorprothixene as a reference ligand. Molecular docking of compounds **1**–**44** was performed using the SP (standard precision) protocol of Glide. 50 poses were generated for each ligands. The selected docking poses were used for CoMFA alignment. PyMol v. 0.99 was used for visualization of results.

### Molecular dynamics

Molecular dynamics studies of selected ligand-receptor complexes were performed using Desmond v. 3.0.3.1 (Bowers et al. 2006) as described previously (Kaczor et al. 2014; Jozwiak et al. 2014; Kaczor et al. 2016a, c). The complexes were inserted into POPC (1-palmitoyl-2-oleoyl-sn-glycero-3-phosphocholine) membrane, hydrated and ions were added to neutralize protein charges and then to the concentration of 0.15 M NaCl. The complexes were minimized and subjected to MD first in the NVT ensemble for 1 ns and then in NPT ensemble for 20 ns with the restrictions on the protein backbone in each case. The production run was performed in NPT ensemble with no restrictions for 50 ns. Analysis of molecular dynamics simulations was performed with application of Schrödinger suite of software tools.

### CoMFA studies

Compounds were divided into the training set (40 compounds) and the test set (4 compounds). For constructing 3D-QSAR models, the compounds should span at least four orders of activity magnitude and be well proportioned in each activity magnitude (Yuan et al. 2014). The activity of the compounds was published elsewhere (Xiao et al. 2014). The AC_50_ (nM) values were converted into pAC_50_, which were applied as dependent variables for subsequent 3D-QSAR analyses. The pAC_50_ of compounds with AC_50_ above 77 μM was arbitrarily assigned the value of 3.

Molecular alignment is the most sensitive factor which has a significant effect on the 3D-QSAR models (Yuan et al. 2014). In this study, by identification of the binding conformations of the compounds, molecular alignment was obtained through molecular docking. Thus, all the molecules were well aligned in the binding site of the dopamine D_2_ receptor for developing 3D-QSAR model.

CoMFA model was developed applying the QSAR module in Sybyl v. 2.1. The standard Tripos force field was used for CoMFA analysis with Gasteiger-Hückel point charges and the default sp^3^ carbon probe with point charge +1.0 (Kaczor et al. 2015). The optimal number of components was designated so that Q^2^ was maximal and the standard error of prediction was minimal (Kaczor et al. 2015).

PLS analysis was applied to linearly correlate the CoMFA fields to the pAC_50_ activity values. The cross-validation analysis was performed using the leave-one-out (LOO) method, in which one compound is removed from the data set, and its activity is predicted using the model derived from the rest compounds of the data set (Kaczor et al. 2015). The model resulting in the highest *Q*^2^, optimum number of components (ONC), and the lowest standard error of prediction were taken for further analysis. In addition, the statistical significance of the model was described by the standard error of estimate (SEE) and probability value (*F* value) (Kaczor et al. 2015).

The predictive capability of the 3D-QSAR model was evaluated with the external test set of 4 compounds. The test set molecules were also optimized and aligned in the same manner as described above, and their activities were predicted using the developed model.

## Results and discussion

### Homology modeling

**Fig. 1:**
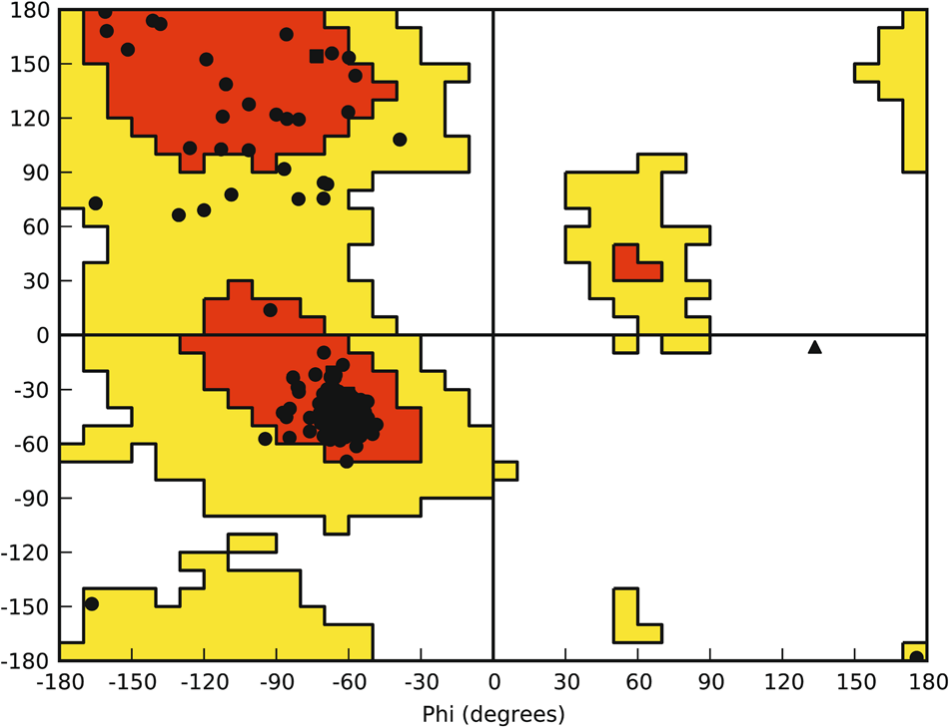
The Ramachandran plot for the D_2_ receptor homology model (Kaczor et al. 2016b)

Homology model of the human dopamine D_2_ receptor (P14416) in inactive conformation and in complex with an antagonist eticlopride was built using homology modeling with Modeler 9.10 (Webb and Sali 2014) and X-ray structure of the dopamine D_3_ receptor in complex with eticlopride (PDB ID: 3PBL) (Chien et al. 2010) as a template as previously described (Kaczor et al. 2016a, b, c). The sequence identity between the template and the target was 79% and the sequence similarity was 90%. The stereochemical quality of the obtained homology model of dopamine D_2_ receptor is confirmed by the respective Ramachandran plot (Fig. 1). High sequence identity and similarity of the template and the target determine the high quality of the homology model, the credibility of resulting docking poses and the obtained CoMFA model. Moreover, this homology model of the human dopamine D_2_ receptor was used for structure-based virtual screening (Kaczor et al. 2016b). In that study, from 21 compounds investigated in vitro we identified ten dopamine D_2_ receptor ligands (47.6% success rate, among them the dopamine D_2_ receptor antagonists as designed) possessing additional affinity to other receptors tested, in particular to 5-HT_2A_ receptors. The affinity (*K*_i_) of the identified compounds ranged from 58 nM to about 24 µM. Importantly, we found one dopamine D_2_ receptor antagonist that did not have a protonatable nitrogen atom, which is a key structural element of the classical D_2_ pharmacophore model necessary to interact with the conserved Asp(3.32) which is rather unusual in structure-based virtual screening. This compound had over 20-fold binding selectivity for D_2_ receptor compared to D_3_ receptor and was also selective over other receptors tested. These findings further confirm the quality of the constructed dopamine D_2_ receptor homology model.

Homology model of dopamine D_2_ receptor in inactive conformation was also built by Malo et al. (2012), Yap et al. (2012), and Duan et al. (2015) using β_2_ adrenergic receptor as a template. Sukalovic et al. (2013) used D_3_ receptor as a template while Kołaczkowski et al. (2013) compared models built on both templates.

### The studied compounds

The studied dopamine D_2_ receptor antagonists **1**–**44** accompanied by their activity (experimental and predicted) are presented in Table 1.

**Table 1:** The investigated dopamine D2 receptor antagonists with the experimental and predicted pAC50 values

Compound number	2D structure	pAC_50_ Exp	pAC_50_ Pred	Residual
Training set				
**1**		7.25	6.66	0.59
**3**		7.15	6.78	0.37
**5**		7.05	6.71	0.34
**6**		7.05	6.84	0.21
**7**		7.05	7.04	0.01
**8**		6.95	6.78	0.17
**9**		6.95	6.91	0.04
**10**		6.95	7.06	−0.11
**11**		6.85	7.10	−0.25
**12**		6.85	6.71	0.14
**14**		6.85	6.93	−0.08
**15**		6.75	6.57	0.18
**16**		6.65	6.49	0.16
**17**		6.65	6.72	−0.07
**18**		6.65	6.73	−0.08
**19**		6.65	6.79	−0.14
**20**		6.55	6.81	−0.26
**21**		6.55	6.30	0.25
**22**		6.55	6.45	0.1
**23**		6.55	6.60	−0.05
**24**		6.45	6.34	0.11
**25**		6.45	6.65	−0.2
**26**		6.45	6.20	0.25
**27**		6.45	6.76	−0.31
**28**		6.45	6.67	−0.22
**29**		6.45	6.49	−0.04
**30**		6.45	6.46	−0.01
**31**		6.35	6.46	−0.11
**32**		6.35	6.60	−0.25
**33**		6.25	6.31	−0.06
**34**		6.25	6.35	−0.1
**35**		6.15	6.04	0.11
**37**		6.15	6.26	−0.11
**38**		6.05	6.04	0.01
**39**		5.80	6.01	−0.21
**40**		5.75	6.00	−0.25
**41**		5.35	5.43	−0.08
**42**		5.05	5.14	−0.09
**43**		3.00	3.02	−0.02
**44**		3.00	2.93	0.07
Test set				
**2**		7.15	6.92	0.23
**4**		7.05	6.75	0.3
**13**		6.85	6.76	0.09
**36**		6.15	6.24	−0.09

### Molecular docking

**Fig. 2:**
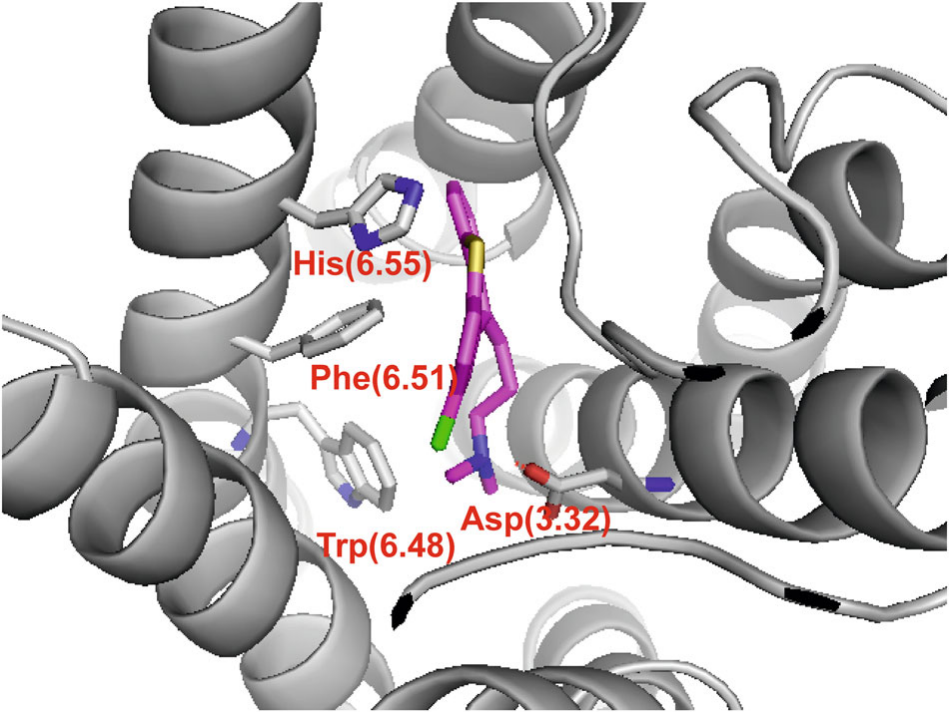
The reference ligand, chlorprothixene docked into the orthosteric site of dopamine D_2_ receptor homology model. Key interaction of the protonatable nitrogen atom of the ligand with the conserved Asp(3.32) (presented as sticks) is shown as red dashed lines. Other important residues, Trp(6.48), Phe(6.51) and His(6.55) also shown as sticks. Transmembrane helices colored in gray. Hydrogen atoms not shown for clarity (color figure online)

A reference ligand, chlorprothixene, was docked to the homology model of dopamine D_2_ receptor using the standard precision (SP) protocol of Glide from Schrödinger suite of software. The selected docking poses were refined using induced-fit docking approach of Schrödinger suite of software. The final docking pose was identified by visual inspection among the poses where the protonatable nitrogen atom of the ligands interacted with the conserved Asp(3.32) of the receptors. The docking pose of chlorprothixene is shown in Fig. 2. It can be seen that a key interaction for this ligand is an electrostatic interaction between the protonatable nitrogen atom of the ligand and Asp(3.32). Moreover, Trp(6.48), Phe(6.61), and His(6.55) were also found to be important for binding of the ligand.

**Fig. 3:**
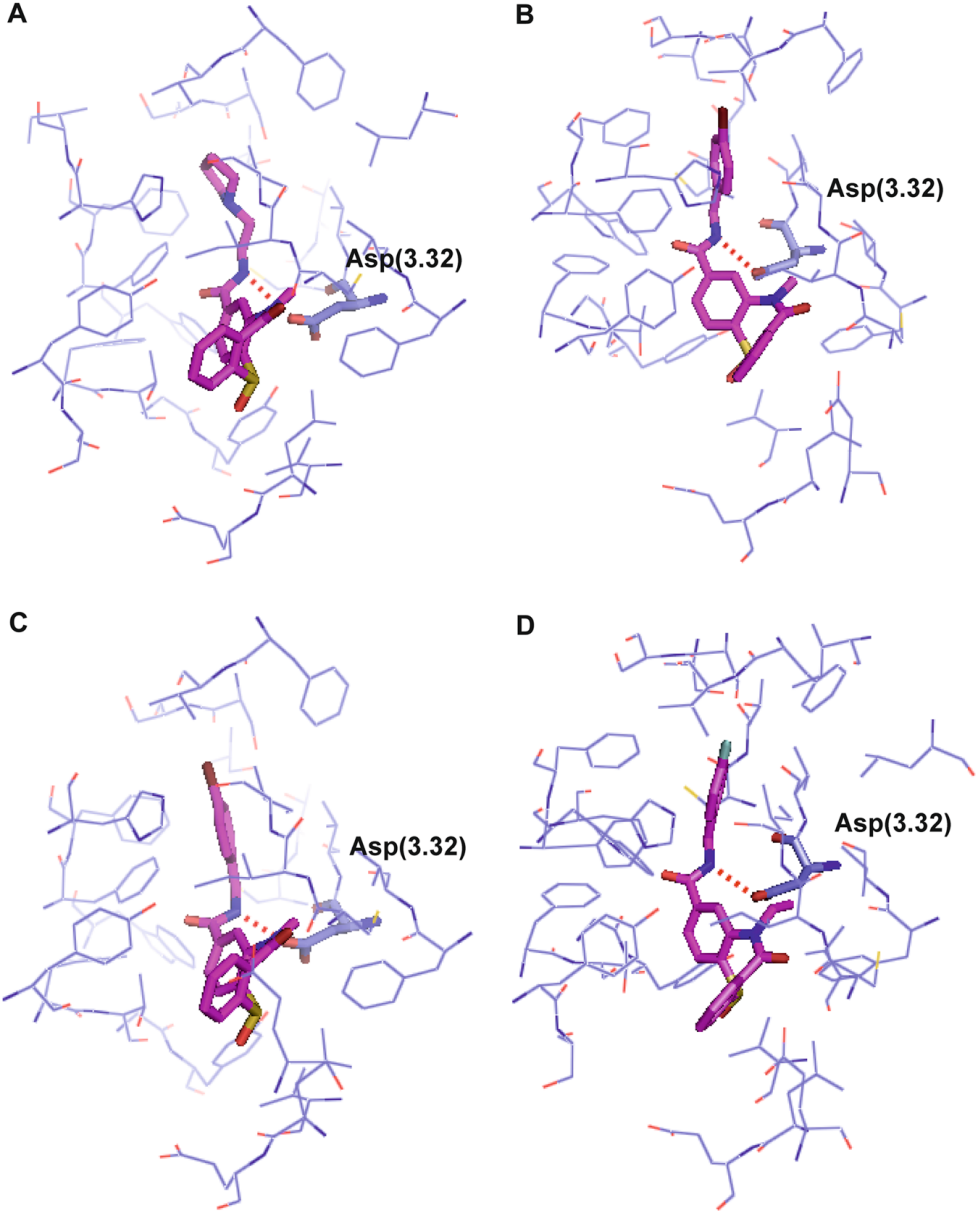
Compounds 2 (**a**), 3 (**b**), 5 (**c**), and 6 (**d**) docked to the orthosteric site of the dopamine D_2_ receptor homology model. Ligands shown as stick with carbon atoms colored in magenta. Protein shown in wire representation with carbon atoms colored in purple. Conserved Asp(3.32) shown as sticks. Hydrogen bonds presented as red dashed lines. Hydrogen atoms not shown for clarity (color figure online)

The studied ligands **1**–**44** (Table 1) were docked to the orthosteric site of the dopamine D_2_ receptor homology model using chlorprothixene-based grid file and Glide of Schrödinger suite of software. The final poses of selected compounds are presented in Fig. 3. In the case of all ligands the key interaction is the hydrogen bond between the amide nitrogen atom of the ligand and the conserved Asp(3.32). Thus, instead of lack of an important pharmacophoric feature, the studied ligands are able to maintain the main contact with the receptor, typical for dopamine D_2_ receptor orthosteric ligands. A similar binding mode was proposed earlier for already mentioned dopamine D_2_ receptor antagonist without a protonatable nitrogen atom identified in virtual screening (Kaczor et al. 2016b).

### Molecular dynamics

**Fig. 4:**
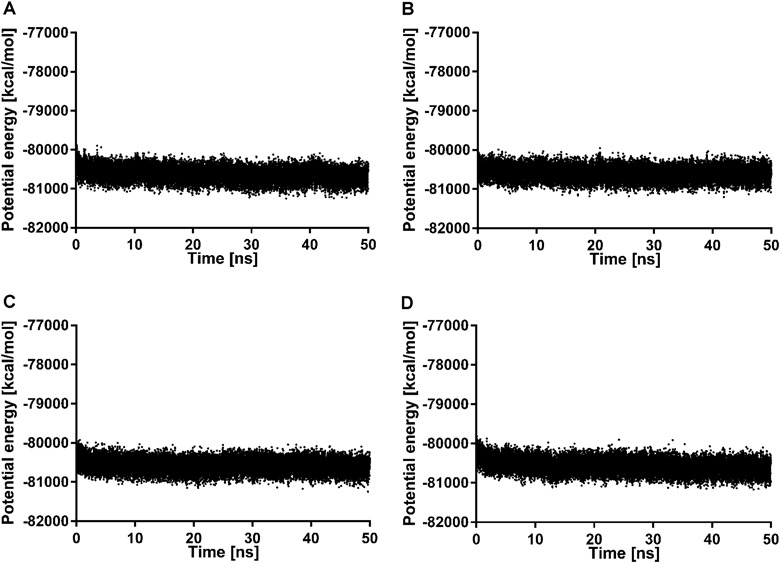
Changes of potential energy during 50 ns molecular dynamics simulation for dopamine D_2_ receptor in complex with 2 (**a**), 3 (**b**), 5 (**c**), and 6 (**d**)

**Fig. 5:**
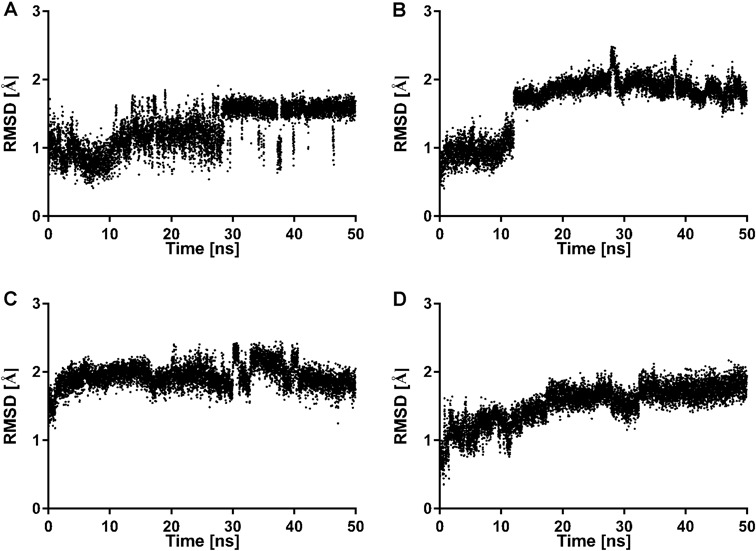
Changes of ligand RMSD during 50 ns molecular dynamics simulation for dopamine D_2_ receptor in complex with **2** (**a**), **3** (**b**), **5** (**c**), and **6** (**d**)

The ligand-receptor complexes were subjected to molecular dynamics with Desmond in order to check their stability. The potential energy changes (Fig. 4) confirm that the simulations were well-equilibrated. The ligand RMSD for selected ligand-receptor complexes is presented in Fig. 5. It can be concluded that the studied ligands are stable in the orthosteric pocket of the dopamine D_2_ receptor which is confirmed by ligand RMSD value below 2.5 Å.

**Fig. 6:**
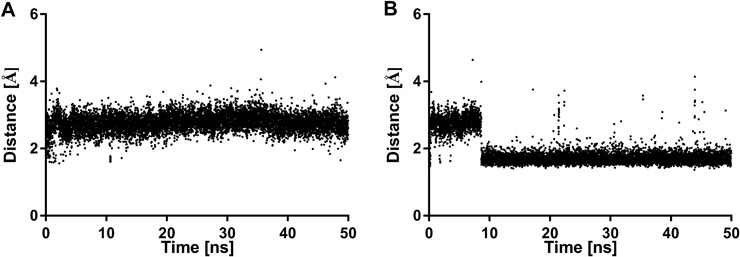
The changes in the ionic lock distance between Arg(3.50) and Glu(6.30) during 50 ns simulations: **a** dopamine D_2_ receptor apo form; **b** dopamine D_2_ receptor in complex with compound **3**

Furthermore, the effect of the studied ligands on the stabilization of the dopamine D_2_ receptor inactive conformation was investigated and compared with the simulations of the apo form of the receptor (Fig. 6). In the inactive state of family A GPCRs there is a strong intramolecular interaction between Arg(3.50) of the conserved (D/E)RY motif in TM3 and residues Glu(6.30) in TM6 (Trzaskowski et al. 2012). Thus, the changes in distance between these ionic lock residues during 50 ns simulations were analyzed. In case of the dopamine D_2_ receptor apo form the distance between Arg(3.50) and Glu(6.30) is stable around 3 Å (Fig. 6a). However, for compound **3** the distance between Arg(3.50) and Glu(6.30) is about 3 Å for the first 10 ns of simulations and then is stabilized below 2 Å (Fig. 6b). This effect is typical for GPCR antagonists and was earlier reported for the dopamine D_2_ bivalent antagonists (Kaczor et al. 2016a).

### Molecular alignment

**Fig. 7:**
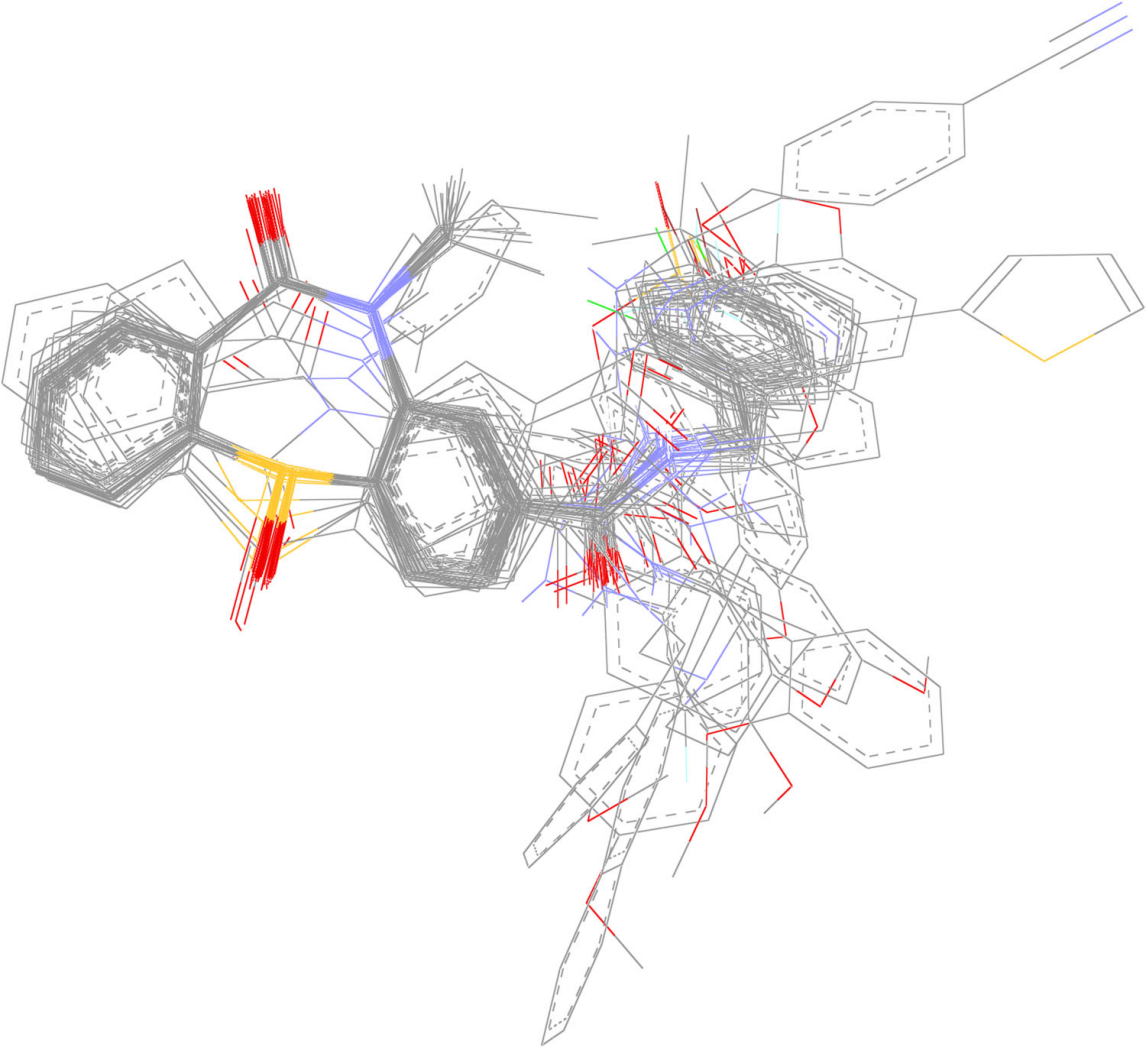
Alignment of **44** compounds in the training and test sets based on the molecular docking results to dopamine D_2_ receptor orthosteric binding site. Non-polar hydrogen atoms omitted for clarity

The quality of 3D-QSAR models depends on the molecular alignment because the compound activities strongly correlate with different substitutions on a specific point in the same compound series (Yuan et al. 2014; Kaczor et al. 2015). The common substructure has been extensively applied as a base for molecular alignment (Verma et al. 2010; Damale et al. 2014). However, better results can be obtained when the 3D-QSAR models could be built and verified on the active conformations of training and test set compounds, in particular when similar ligands occupy different binding poses in the binding site (Urniaż and Jóźwiak 2013). The molecular alignment of the compounds from the training and test sets is shown in Fig. 7. The alignment using docking conformations will facilitate understanding the contour maps of the models in a structure-based manner (Yuan et al. 2014; Kaczor et al. 2015).

### CoMFA statistics

**Fig. 8:**
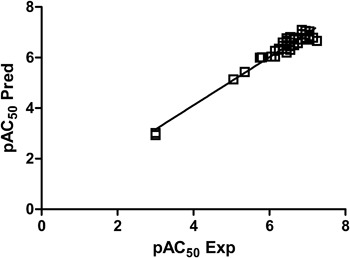
The experimental versus predicted pAC50 values for the training set

The 3D-QSAR CoMFA model was built using Sybyl-X v. 2.1. The CoMFA model gave a cross-validated coefficient *Q*^2^ of 0.63 with an optimal number of components of 4, *R*^2^ of 0.95 and *F* value of 174.133. The standard error of estimate was 0.207. These statistical parameters indicate that the CoMFA model is statistically significant. A model for which *R*^2^ is above 0.9 and *Q*^2^ is above 0.4 is usually considered to be predictive (Wang et al. 2009). The respective field contribution parameters were 62.9% for the electrostatic field and 27.1% for the steric field descriptor. Experimental and predicted AC_50_ values are presented in Table 1. It can be seen that they are not deviated significantly from each other (not more than 0.3 logarithmic unit for the majority of compounds, with the exception of compound **1** from which was slightly under-predicted). Figure 8 shows a very good correlation between the experimental and computed IC_50_ values for the training set.

### Validation of CoMFA model

**Fig. 9:**
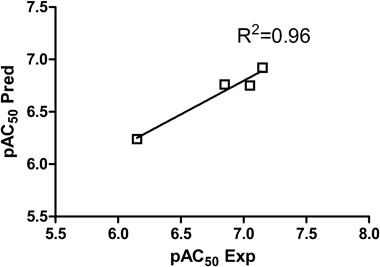
The experimental versus predicted pAC_50_ values for the test set

The obtained CoMFA model was validated by the external test set of four compounds (10% of the number of training set compounds). The *R*^2^ of test set was 0.96 so it was close to *R*^2^ of the training set which confirms the good predictability of the CoMFA model (Fig. 9) (Golbraikh and Tropsha 2002). Thus, the activities of all the test set compounds were correctly predicted.

### Contour map

**Fig. 10:**
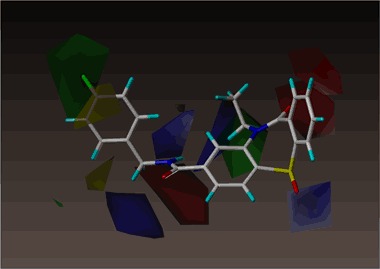
CoMFA steric and electrostatic contour fields. Fields drawn with 90/10 proportion of favorable and unfavorable interactions. The most active compound **1** shown

Figure 10 shows the steric and electrostatic contour maps gained via CoMFA modeling. Steric contour maps gave the information about the spatial volume of substituted groups on different positions. There were two green contour regions located in the active site, meaning that bulky groups were favored. There is a green region near the nitrogen atom of the tricyclic dihydrodibenzo[b,f][1,4]thiazepine system meaning that some substituents may be beneficial in this position. Indeed, the methyl group in this position leads to compounds with better potency than compounds bearing this position not alkylated. However, ethyl, propyl and benzyl moiety lead to decreased potency with increasing substituent size. Another position for a bulky group was identified in the para position of the benzyl group found as primary amide substituent in several compounds. The nature of the substituent in this position does not have an effect on the compound potency. Both the electronic and steric nature of substituents is tolerated. Meta substitution is less favored and ortho substitution is not tolerated (two yellow regions in the contour map). Two red contour regions near carbonyl groups indicate that more negative charge is favored here. As negative charge is also connected with H-bonding acceptors this would most probably also indicate H-bonding acceptors to be favored. Furthermore, the S-oxide group is connected with blue contour region meaning that more positive (or less negative) charge and, correspondingly, H-bond donors are favored and negative charge /H-bond acceptors are disfavored.

## Conclusion

We built a statistically valid CoMFA model for the dopamine D_2_ receptor antagonists without a protonatable nitrogen atom. The model was based on molecular alignment derived from molecular docking to the orthosteric site of the dopamine D_2_ receptor homology model. In spite of having no protonatable nitrogen atom which is a key element of the classical pharmacophore model, the studied compounds are able to interact with the conserved Asp(3.32) thanks to their amide nitrogen atom. The constructed CoMFA model was characterized with the following statistics: *R*^2^ = 0.95, *Q*^2^ = 0.63. The quality of the CoMFA model was confirmed by high value of *R*^2^ of the test set, equal 0.96. The CoMFA model allowed to identify two regions where bulky substituents are favored and two regions where bulky substituents are not favorable Two red contour regions near carbonyl groups were identified meaning that negative charge would be favored here while the S-oxide group is connected with blue contour region meaning that positive charge is favored in this position. These findings may be applied for further optimization of the studied compound series. Moreover, the studied compounds stabilize the receptor inactive conformation through the effect on the ionic lock which is typical for GPCR antagonists.
